# When “altering brain function” becomes “mind control”

**DOI:** 10.3389/fnsys.2014.00202

**Published:** 2014-10-14

**Authors:** Andrew Koivuniemi, Kevin Otto

**Affiliations:** ^1^Indiana University School of MedicineIndianapolis, IN, USA; ^2^Weldon School of Biomedical Engineering, Purdue UniversityWest Lafayette, IN, USA; ^3^J. Crayton Pruitt Family Department of Biomedical Engineering, University of FloridaGainesville, FL, USA

**Keywords:** philosophy of mind, ethics, neurosurgery, deep brain stimulation, psychiatry

## Abstract

Functional neurosurgery has seen a resurgence of interest in surgical treatments for psychiatric illness. Deep brain stimulation (DBS) technology is the preferred tool in the current wave of clinical experiments because it allows clinicians to directly alter the functions of targeted brain regions, in a reversible manner, with the intent of correcting diseases of the mind, such as depression, addiction, anorexia nervosa, dementia, and obsessive compulsive disorder. These promising treatments raise a critical philosophical and humanitarian question. “Under what conditions does ‘altering brain function’ qualify as ‘mind control’?” In order to answer this question one needs a definition of mind control. To this end, we reviewed the relevant philosophical, ethical, and neurosurgical literature in order to create a set of criteria for what constitutes mind control in the context of DBS. We also outline clinical implications of these criteria. Finally, we demonstrate the relevance of the proposed criteria by focusing especially on serendipitous treatments involving DBS, i.e., cases in which an unintended therapeutic benefit occurred. These cases highlight the importance of gaining the consent of the subject for the new therapy in order to avoid committing an act of mind control.

## Introduction

The use of deep brain stimulation (DBS) technology for the treatment of psychiatric disorders is one of the most promising and rapidly evolving areas of neurosurgical research (Abelson et al., 2005; Mayberg et al., 2005; Lozano and Lipsman, 2013). Nonetheless, in treating diseases of the mind by directly altering the brain’s functioning, neurosurgeons, neurologists, psychiatrists, and neuro-engineers run the risk of having this effort interpreted as “mind control”. The purpose of this paper is to address that specific concern in the context of DBS as it is currently practiced and studied by providing a definition of “mind control” that applies to DBS. That is, it is not intended to account for the neurosurgeons staffing the wards of philosophical thought experiments, whose powers to monitor and manipulate the brain and their patients’ actions know no limits (Frankfurt, 1969). Therefore, this paper seeks to cover adult patients who have given informed consent for the treatment of their psychiatric or neurologic illness.

By narrowing the scope of the article, we hope to maximize its relevance while minimizing distracting (though philosophically interesting) cases. It is important to point out that the conditions under discussion (adults, undergoing treatment, who are capable of informed consent—including patients in states such as locked-in syndrome) describe nearly all individuals currently receiving DBS with the exception of those treated for persistent vegetative state who lack the capacity to do anything, including the ability to provide consent (Yamamoto et al., 2010).

While the phrase “mind control” appears in the contemporary literature discussing advances in DBS, it is often brought up dismissively (Fins et al., 2009) or to catch the reader’s attention (Horgan, 2004) but never with an accompanying formal definition. This is surprising, especially given a sophisticated and robust ethics literature on DBS and psychiatry that deals with related topics such as autonomy and informed consent (Bell et al., 2009; Clausen, 2010), authenticity (Kraemer, 2013), enhancement (Earp et al., 2014), and paternalism (Sjöstrand and Juth, 2014) as well as unintended side effects of stimulation which alter personality (Synofzik and Schlaepfer, 2008) and the way in which DBS can influence patients’ perceptions of their identity (Lipsman et al., 2009). Common features in this literature are an agreement that autonomy is one of the key features that must be preserved in the ethical practice of DBS and that this can been accomplished in psychiatric patients through the practice of informed consent (Dunn et al., 2011).

Beyond the contemporary neuro-ethics literature, mind control has been the subject of numerous books and articles. One of the most thorough accounts of mind control in the context of electrical stimulation of the human mind appears in Elliot Valenstein’s aptly titled *Brain Control* (Valenstein, 1973). While Valenstein never supplies a formal definition of mind control, his primary argument focuses on discrediting the notion that a subject’s thoughts, choices or actions could be manipulated through electrical stimulation of the brain by giving a detailed account of its known capabilities and limitations. Other discussions of mind control tend to have focused on psychopharmacologic methods or behavioral methods of altering brain function, such as those employed in the Central Intelligence Agency’s MKULTRA program (Senate, 1977).

Possibly the richest source of accounts of mind control is not in the formal academic literature but in the online accounts of individuals who claim to have witnessed acts of mind control or who claim to be the target of mind control. In 2006 Bell et al. provided a formal textual analysis of 10 characteristic examples. Though the authors make it clear that they take these narratives as signs of a delusional disorder and their analysis focused primarily on the social network of the reports, they managed to highlight several themes which the accounts shared. These shared features help to establish an intuitive basis for what people believe qualifies as mind control. The accounts often focused on: (1) an authoritarian organization, such as “the police,” “the Dutch government,” or “freemasonic intelligence agencies;” (2) employing some tool to augment brain function, such as a “frequency weapon,” “brain implant” or “network of transmitters,” in order to; (3) alter the subject’s thoughts or actions; (4) without the subjects consent.

In proposing our criteria for mind control we retained and formalized all of the common themes of the internet accounts with the exception of the authoritarian organization. The authoritarian element was dropped because the authors saw no reason to exclude individuals acting alone from being capable of committing an act of mind control. This is especially true in the context of DBS where typically only one person or a few people are responsible for the management of the treatment. Therefore, we are proposing the phrase “mind control” be used to describe instances when researchers or clinicians using DBS intentionally alter patients’ behavior without consent and define those instances using the criteria below.

After stating our formal criteria, we explain why the criteria are limited to the subject’s behavior and neutral with regards to the subject’s mental events during the act of mind control. Then we provide test cases, which we argue intuitively do and do not qualify as mind control and are correctly included and excluded by the proposed criteria respectively. Next, we apply the criteria to a non-obvious case of mind control. Finally, we conclude with a discussion of mind control in the context of serendipitous therapy, i.e., cases where an individual sees a therapeutic effect for a psychiatric illness for which he or she did not give consent to have treated, such as in a patient treated with DBS for anxiety who saw a remission of his alcoholism. We argue that in such cases one should gain the individual’s explicit consent for the treatment of the serendipitously improved co-morbid illness or else one would qualify as committing an act of mind control.

## Criteria of mind control

Alteration of the brain’s functioning through direct stimulation (either activation or suppression of action potentials) within the subject’s brain qualifies as mind control when it meets all of the following three criteria:

*Result Criterion*: Direct alteration of the brain’s function must result in a behavioral change in the subject.

*Consent Criterion*: The behavioral change does not need to be against the expressed will of the patient. The change must simply have taken place without the subject’s consent.

*Intent Criterion*: The behavioral change must have been the goal or the purpose of the person or the group controlling the DBS. It cannot be an accident or an unintended consequence, including side effects, of the stimulation.

In summary, mind control must alter the patient’s behavior in an observable way without the subject’s consent and must be enacted for that purpose.

## Limiting mind to behavior

The above criteria rest on an assumption that the ultimate purpose of “mind control” is to modify the behavior of an individual, and the word “mind” is used in a folk psychology manner to describe the intuitive mechanism of the control (Dennett, 1982). It is important to spell out the definition of “mind control” in the context of behavior because that is the relevant way DBS is currently employed. This is because neurosurgeons and neurologists cannot make perfectly reliable *a priori* guesses about what effect a given instance of DBS will have on a given patient. They must therefore rely entirely on their observations of the patients’ behaviors, which include their patients’ reports.

To understand this point, consider that neurosurgeons have a great deal of information about what parts of the brain are associated with certain faculties, such as the formation and comprehension of speech, sensation of touch over the body, execution of intended movement, and sight. Further, they know that the destruction of these regions will leave the patient with a deficit so protecting them during surgery is one of the surgeon’s highest priorities. However, the surgeons cannot predict exactly where these regions are located in specific patients based on previous studies alone (Penfield and Perot, 1963; Kim et al., 2009). Therefore, some neurosurgical cases are performed with the patient awake so that he or she can report the sensations he or she experiences when the neurosurgeon applies electric current to the brain region of interest. Based on the patient’s reports, the surgeon will individualize his approach in order to resect the pathological tissue while sparing the functionally important, so called eloquent, cortex. If the procedure were performed without the patient’s behavioral feedback there would be a very high probability that an important cortical region would be damaged leaving the patient with a neurological deficit (Penfield and Boldrey, 1937).

The same type of procedure is also essential to the practice of DBS. For example patients must be closely observed intra-operatively for behavioral signs, such as a reflexive smile, in order for the surgical team to determine the effect of stimulation (Okun et al., 2004; Haq et al., 2011). Once the electrode and stimulator are implanted, specially trained neurologists adjust the stimulation parameters and closely observe the effect on the patient’s symptoms (Volkmann et al., 2006). Finally, patients must be closely followed during treatment for signs of cognitive decline (Parsons et al., 2006), mood disorders (Bejjani et al., 1999; Kulisevsky et al., 2002), or other, sometimes serendipitous, behavioral changes (Kuhn et al., 2007). In summary, the use of DBS relies entirely on the patient’s behavior as the sole feedback mechanism for targeting the electrode as well for modifying the stimulation parameters in order to achieve the desired effect. Because the person or persons controlling the DBS rely on observation of behavior, any instance of mind control using DBS would necessarily rely entirely on the subject’s behavior. Therefore, a practical definition of mind control can be limited solely to behavior without directly addressing metaphysical questions related to the mind itself.

## Obvious test cases

Having proposed the criteria for mind control, it is important to test them. This is best done by asking whether the criteria account for cases of obvious mind control while excluding cases that are obviously not mind control.

For a clear example of mind control, we must (fortunately) look beyond the current practice of DBS into its murkier past. One such case was published in 1963 in the journal *Science* by a psychosurgery group working under Dr. Robert Heath at Tulane University (Bishop et al., 1963). This article detailed a “self-stimulation” experiment in which a 35 year old man was implanted with electrodes in eight different brain structures, including in the head of the caudate, the septal area, and the amygdala. These electrodes were labeled by researchers as either “rewarding” or “aversive” and the subject was given a lever and a button which, when operated, would activate one of the electrodes. As the experiment proceeded, the researchers varied the electrodes which the lever and button activated and also varied the stimulation parameters delivered through the electrodes.

This experiment was based on studies previously done in rats, cats, dogs, goats, monkeys, and bottle nosed dolphins (Olds, 1962) which had shown that the animals’ behavior could be predictably controlled by placing stimulating electrodes into “rewarding” and “aversive” regions of the brain and then correlating stimulation through the electrodes to elements of the animals’ environment. Therefore, the researchers had good reason to anticipate specific behavioral responses in the human subject. Further, at no point do the authors say that the subject, who was referred to as “clearly nonnormal,” gave consent for the experiment or understood why the experiment was conducted.

Looking back to the proposed criteria for mind control, we see that this case satisfies all three. First, electrical stimulation of the brain was employed in a manner that clearly influenced the subject’s behavior, satisfying the *Result Criterion*. Second, at no point did the authors state that the patient gave consent to have his behavior manipulated in this manner, satisfying the *Consent Criterion*. Finally, the behavior change was anticipated by the researchers controlling the stimulation of the subject’s brain, satisfying the *Intent Criterion*.

Next, we must ask is there an example of altering brain function which obviously is not mind control and, also, is correctly excluded by the *Result, Consent*, and* Intent Criteria*? Consider the treatment of essential tremor with DBS. It is safe, effective, and has been approved by the FDA (Koller et al., 2001). It is believed to work through altering the function of the brain (more specifically by causing a reversible, functional lesion (Grill et al., 2004) in a malfunctioning part of the brain), ultimately permitting the patient to accomplish routine daily activities free from the violent hand tremors that are the hallmark of the disease. This relief of symptoms is the direct result of the electrical pulses in the brain, which alter its standard pattern of firing; however, it is not an instance of mind control.

Why is DBS for the treatment of essential tremor not an example of mind control? After all, it could be argued that one is altering the behavior of the patient’s hands, from a tremulous grasp to a stable grip, and that this was explicitly the purpose of the individual programing the DBS device. However, while this example meets the requirements of the *Result Criterion* as well as the *Intent* *Criterion*, it fails to meet the *Consent Criterion* because in all cases of DBS for essential tremor, all patients give consent for stimulation with the explicit desire to see this behavioral change. Interestingly, DBS for essential tremor could be thought of as “mind freedom,” as opposed to “mind control” because, instead of preventing the patient from carrying out a desired behavior or forcing an undesired behavior, it allows the patient to act on his choices with less difficulty.

The same argument also holds for DBS treatments of psychiatric diseases like depression (Lozano et al., 2008). One might make the argument that being a psychiatric disease, depression is classically described as a disease of the mind. Therefore, if one can control the patient’s disease one must be controlling the patient’s mind, i.e., committing an act of mind control. The proposed criteria would exclude this case of mind control because, as in the case of DBS for the treatment of essential tremor, the effect on the patient was with the patient’s consent, and, thus, it fails the *Consent Criterion*.

## Non-obvious test case

While it is important that the criteria capture one’s intuition, they should also go beyond and clarify murkier territory. The criteria should be able to help one examine non-obvious cases and arrive at a reasoned judgment about their status as mind control or as non-mind control. Thus, the criteria above are especially useful when attempting to identify borderline instances of mind control.

Turning again to the past, consider the following case of an experiment conducted by Jose Delgado and his collaborators Drs. Obrador and Martin-Rodriguez into the stimulation of the caudate nucleus of an epileptic patient:

As shown by direct observation and by analysis of the record, within 30 s after application of caudate stimulation there was a significant change in the patient’s mood. During controls, he was reserved, his conversation was limited and he was concerned about his illness. After caudate stimulation, his spontaneous verbalization increased more than twofold and contained expressions of friendliness and euphoric behavior which culminated in jokes and loud singing in a gay *cante jondo* style, accompanied by tapping with his right hand, which lasted for about 2 min. The euphoria continued for about 10 min and then the patient gradually reverted to his usual, more reserved attitude. This increase in friendliness was observed following three different stimulation sessions of the caudate, and did not appear when other areas were tested (Valenstein, 1973).

In the above description, the researchers are attempting to correct the patient’s epilepsy with the use of electrical current. In testing one of their hypothesized targets, they managed to elicit a strong behavioral effect. The patient’s attitude changed from quiet reserve to expressive joviality, i.e., the researchers significantly altered the patient’s behavior and in doing so satisfied the *Result Criterion*, as well as the *Consent Criterion* because they did not have the patient’s consent to alter his behavior in this manner. At this point one could argue, correctly, that this was an accident. The experimenters had no *a priori* knowledge that the patient would respond to stimulation in this fashion so it could not have been their intention to do so; thus, they failed to satisfy the *Intent Criterion*.

The essential issue arose when the stimulation was repeated, three different times, without any documentation that the patient wanted to have his personality manipulated in this manner. While this might, at first, seem like nit picking, it is important to appreciate that the experimenters now had reason to believe that the behavior of the individual would be affected in a specific way. When they activated the stimulation and produced the anticipated effect, it was purposeful. In this way, the experimenters fulfilled the *Intent Criterion*. As in the case of a schizophrenic patient subjected to the self-stimulation experiment above, it seems clear that the researchers’ motivation was intellectual curiosity and not malice. Nevertheless, both of these cases demonstrate that malice is not necessary for mind control.

## Serendipity and mind control

The above case raises a critical question with regard to several recently published studies in which subjects received DBS in an effort to treat one illness, but instead saw serendipitous improvement in a comorbid psychiatric illness. One serendipitous discovery was reported by Kuhn et al. (2007) who attempted to treat a man with anxiety disorder by placing DBS electrodes into his nucleus accumbens, a major component in the reward circuit of the mammalian brain. While the patient’s anxiety did not improve, he did see significant remission in his alcohol dependency, leading the group to propose the target as a potential treatment for alcoholism and addiction.

A second example comes from Hamani et al. (2008) who used DBS of the hypothalamus in an effort to help control a patient with morbid obesity. Although the patient continued to gain weight (a fact left out of the primary article and only included in the online supplemental materials) he did experience a flashback while receiving intra-operative test stimulation. This led the researchers to do a battery of studies to determine if stimulation to the same area at a lower level, which did not cause a flashback, could improve memory. To the surprise of the researchers, they found a significant increase in the subject’s verbal memory. Based on this finding the authors proposed the anterior fornix (a structure adjacent to the hypothalamus) as a target for the treatment of dementia and began enrolling patients to study it further.

Finally, Israël et al. (2010) describes a case in which a woman was receiving DBS of the subgenual cingulate gyrus (Cg25) for treatment of depression. The authors noted that, although the patient continued to have relapses of major depression, she stopped experiencing symptoms related to a significant comorbid anorexia nervosa. Based on the remarkable improvement the patient experienced, despite her less remarkable improvement for her depression, the authors proposed Cg25 as a target for the treatment of anorexia nervosa.

There are several curious similarities among the cases above. First, the intended effect of DBS was either not seen or was not particularly robust. Second, the serendipitous effect on the comorbid illness (or enhancement of normal faculties in the case of anterior fornix stimulation for memory) was remarkable. Third, based on these cases all authors proposed that the stimulated sites be tested as targets for monotherapy for the responding illness. A final common feature was that at no point did the authors describe the patient receiving informed consent for the managing of the comorbid illness or for enhancing the patient’s faculties (Earp et al., 2014), with DBS. The only paper that commented on informed consent was Hamani et al. which stated:

The procedure was approved by the University Health Network Research Ethics Board, and written informed consent was obtained under the guidance of a hospital ethicist, who served as a consent monitor. The basis of the approval for this man was the refractory nature of the obesity, the exhaustion of reasonable therapeutic alternatives, and the possibility of reducing the health risks of chronic obesity should the intervention prove successful (Hamani et al., 2008).

In the passage above, the authors clearly stated a reasonable approach for obtaining informed consent for the treatment of the patient’s obesity. However, they did not describe receiving the patient’s consent for the use of DBS in order to enhance his verbal memory. Despite not reporting the patient’s informed consent to have his memory augmented, they proceeded to run a battery of tests on the patient’s memory function and, furthermore, did not mention discontinuing the treatment once it became apparent that DBS was not effective for the treatment of obesity.

The above cases raises a critical question: were these examples of “mind control”? The patients had unexpected alterations in their behavior and it appears, based on the descriptions of the cases, that the DBS was continued primarily because of these unexpected results. Further, the authors did not report that they repeated the informed consent process for the serendipitous alteration in the patient’s behavior. The authors of this paper could conjecture that, once the researchers realized the unexpected effect DBS was having on their patient they consulted with him or her and received his or her blessing to continue therapy. Nonetheless, if they (or others) had not secured the consent of their patients for these new treatment indications, then they would be satisfying the *Result* (behavior change) *Consent* (happening without patient’s consent) and *Intent* (behavioral change was the goal of DBS) criteria of mind control. Therefore, it is critical for clinicians and researchers to secure additional consent in the case of serendipitous therapeutic benefit in order to avoid the charge that they are committing an act of mind control.

## Conclusion

We have argued that DBS is not synonymous with mind control; however, if not appropriately safeguarded, patients can be victims of mind control even without malice on the part of those controlling the stimulation, especially in the case of serendipitous treatment of co-morbid psychiatric illnesses. While many instances of mind control are easily identified, there are certain instances where the distinction is more ambiguous. This paper outlines a clear set of criteria to help more effectively and reliably clarify those ambiguous cases. For an act to be considered mind control it must alter the individual’s behavior (*Result Criterion*) without his consent (*Consent Criterion*) and this alteration to the behavior of the individual must be the goal of the person or group controlling the alteration (*Intent Criterion*). Relying on the researchers’ or clinicians’ intuitions alone is not sufficient because those intuitions might easily become clouded such as in the serendipitous discovery of an effect of DBS. It is, therefore, important to note that in cases of serendipitous treatments of psychiatric illness patients also require the explicit consent for the treatment of the co-morbid illness, or else the case would qualify as mind control. It is the intention of the authors to minimize the risk of such accidents by clarifying the underlying concepts.

## Conflict of interest statement

The authors declare that the research was conducted in the absence of any commercial or financial relationships that could be construed as a potential conflict of interest.
